# Effect of simulated hearing loss on automatic speech recognition for an android robot-patient

**DOI:** 10.3389/frobt.2024.1391818

**Published:** 2024-09-02

**Authors:** Jan Hendrik Röhl, Ulf Günther, Andreas Hein, Benjamin Cauchi

**Affiliations:** ^1^ Assistance Systems and Medical Device Technology, Health Services Research, Carl von Ossietzky Universität Oldenburg, Oldenburg, Germany; ^2^ Klinikum Oldenburg AöR, Oldenburg, Germany; ^3^ R&D Division Health, OFFIS e.V., Institute for Information Technology, Oldenburg, Germany; ^4^ Management and Information Systems, Bremerhaven University of Applied Science, Bremerhaven, Germany

**Keywords:** hearing loss simulation, automatic speech recognition, android robot-patient, simulated patient, patient simulation

## Abstract

The importance of simulating patient behavior for medical assessment training has grown in recent decades due to the increasing variety of simulation tools, including standardized/simulated patients, humanoid and android robot-patients. Yet, there is still a need for improvement of current android robot-patients to accurately simulate patient behavior, among which taking into account their hearing loss is of particular importance. This paper is the first to consider hearing loss simulation in an android robot-patient and its results provide valuable insights for future developments. For this purpose, an open-source dataset of audio data and audiograms from human listeners was used to simulate the effect of hearing loss on an automatic speech recognition (ASR) system. The performance of the system was evaluated in terms of both word error rate (WER) and word information preserved (WIP). Comparing different ASR models commonly used in robotics, it appears that the model size alone is insufficient to predict ASR performance in presence of simulated hearing loss. However, though absolute values of WER and WIP do not predict the intelligibility for human listeners, they do highly correlate with it and thus could be used, for example, to compare the performance of hearing aid algorithms.

## 1 Introduction

Worldwide the life expectancy is increasing in most regions (Buskens et al., 2019). As a consequence, despite the decrease in birth rates, the global population is both expanding and aging (Gu et al., 2021). This demographic shift towards an aging population necessitates greater attention to medical care. This care should be adapted to the needs of the elderly, including hearing loss that affects more than half of them (Dalton et al., 2003).

The prevalence of hearing loss is even larger for patients suffering from heart failure or delirium (Morandi et al., 2021; Baiduc et al., 2023). Delirium is a significant neurocognitive disorder that may arise due to a medical condition, a drug-induced psychotic disorder, or following a surgical procedures performed under anesthesia on geriatric patients (Association. and Association., 2013; Devlin et al., 2018; Ely et al., 2004; Ely et al., 2001a; Rudolph et al., 2010; 2009). The Confusion Assessment Method for the Intensive Care Unit (CAM-ICU) is an established assessment for the diagnosis of delirium (Ely et al., 2001b; Guenther et al., 2010). The training of assessment methods, like the CAM-ICU, is complex and time consuming. In addition to the need for a sufficient number of patients with different types of delirium and without delirium, training is only possible on a small scale due to the stress of the patients. An alternative to real patients are standardized human patients/simulated patients (SP) (Barrows, 1968; Pourebadi and Riek, 2022). SPs, i.e., specially trained actors, are considered an effective learning method, but they are scarce and expensive (Tengiz et al., 2022; Cleland et al., 2009). Beyond the value of the SPs, there are significant concerns about comparing the experiences of different training groups. For example, in the evaluation of SPs there is evidence of numerous differences between cases in the behavior of SPs over a number of simulations, and it is simply not possible to compare the experiences of one group with those of another (Austin et al., 2006). As a result of these concerns, educational institutions have a strong interest in the robotic simulation of patient behavior to reduce reliance on patients who are suitable for medical assessment training (Buchanan, 2001; Gaba, 2004).

**FIGURE 1:**
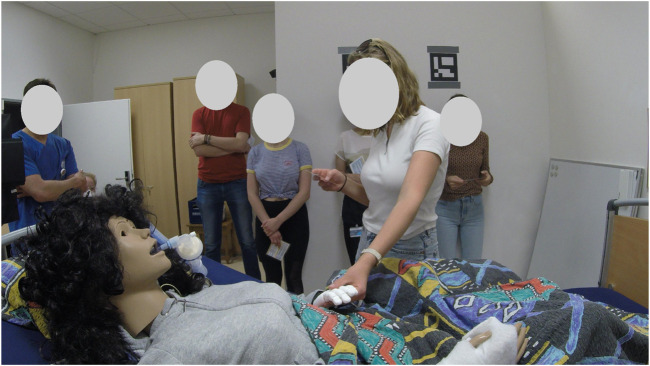
Android robot-patient (ARP) simulating realistic patient behavior.

Robotic systems and android robot-patients (ARPs) have been introduced for teaching purposes (Abe et al., 2018; Tanzawa et al., 2012; Tanzawa et al., 2013; Hashimoto et al., 2013; Pourebadi and Riek, 2022; Gaumard Scientific Company, 2022a; Gaumard Scientific Company, 2022b; CAE, 2022; Haley et al., 2017; Schwarz and Hein, 2023; Röhl et al., 2022; 2023). With the focus on medical dental education, especially the communication and risk management, an ARP was evaluated by using a student’s questionnaire, which showed that 95% of the students recognized the usefulness to train the risk management with the ARP (Tanzawa et al., 2012; Tanzawa et al., 2013). An other ARP, called SAYA, simulates a depressed patient for diagnostic training (Hashimoto et al., 2013). With a focus on nursing procedures and communication with patients, there are various simulators, which can move their head or simulate human facial expressions, vital signs, and specific diseases, and are promising tools for clinical training (Pourebadi and Riek, 2022; Gaumard Scientific Company, 2022a; Gaumard Scientific Company, 2022b; CAE, 2022; Haley et al., 2017). Furthermore, there are already efforts to use high-end robots like AMECA in training and continuing education programs for medical staff with a focus on depression (Schwarz and Hein, 2023). With ongoing work on an ARP (see Figure 1) to simulate a critically ill nonverbal patient, it has already been shown in an initial simulation with an ARP, that it has the ability to reproduce human behavior (Röhl et al., 2022). Since the detection of delirium is important, there have been efforts in simulating patients with and without delirium via an ARPs for the education of medical staff in delirium-assessment methods (Röhl et al., 2023).

As delirium assessment is a verbal (medical experts) to nonverbal (patient) communication, the ARP should be able to listen. Therefore, an automatic speech recognition (ASR) was implemented. ASR is often implemented in robotic simulators using small weight models, typically using Vosk (Glauser et al., 2023; Fadel et al., 2022; Paul et al., 2022). Since elderly patients often present with hearing loss, the effect of hearing loss on ASR performance has to be carefully considered. The evaluation of this impact is the focus of this paper, whose remainder is structured as follows. The used methodology is described in Section 2. This entails the description of the used dataset, of the hearing loss simulation and ASR implementation, and of the considered evaluation metrics. The results, obtained using simulated hearing loss based on audiograms from real listeners, are presented in Section 3 before presenting the conclusions.

## 2 Methods

**FIGURE 2:**
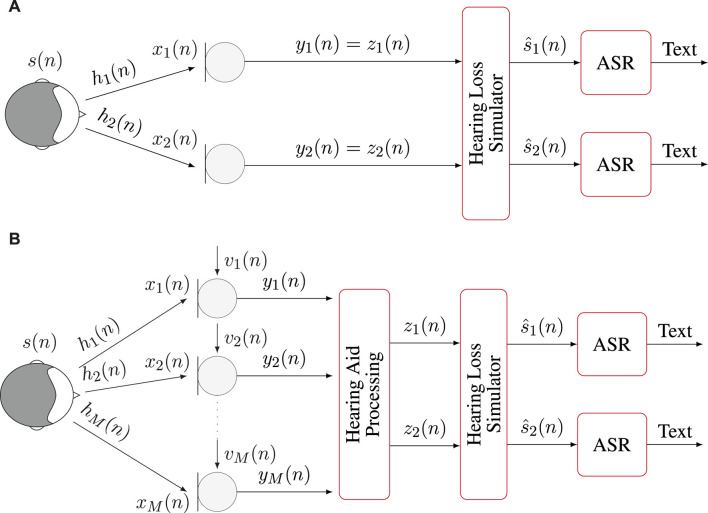
Overview of the signal processing chains used in the experiments. **(A)** Binaural anechoic signals are processed through the hearing loss simulator before applying ASR. **(B)** Multichannel noisy and reverberant signals are enhanced through hearing aid processing before being input to the hearing loss simulator and applying ASR.

The experimental framework presented in this paper had two objectives. First, it aimed at quantifying the impact that hearing loss, simulated using measurements from real listeners, might have on the performance of an ASR system in presence of clean anechoic speech. Second, it aimed at evaluating the joint impact of hearing loss and hearing aid processing on the performance of an ASR system in presence of noisy reverberant speech, i.e., in realistic conditions. The experiments were conducted using data made available as part of the second edition of the clarity prediction challenge (CPC) (Graetzer et al., 2021) and publicly available ASR models to be used with the Vosk toolkit (Shmyrev, 2023). An overview of the signal processing chains used for both objectives is depicted in Figure 2 and summarized in the remainder of this section.

### 2.1 Auditory scene generation

The audio signals from the CPC used in the experiments were generated as follows. Anechoic speech signal s(n) at a sampling frequency $f_{s}=$ 48 kHz, where n denotes the sample index, containing 7 to 10 words and for which text prompts are known, were used as the target signals to be recognized (Graetzer et al., 2022). Input signals $y_{m}\left(n\right)$ were generated described in Equation 1.

$$y_{m}\left(n\right)=\underset{x_{m}\left(n\right)}{\underbrace{s\left(n\right)*h_{m}\left(n\right)}}+v_{m}\left(n\right),$$ (1)

where $h_{m}\left(n\right)$ denotes the room impulse response (RIR) between the source and the m -th of M microphones, $x_{m}\left(n\right)$ denotes the clean reverberant signal and $v_{m}\left(n\right)$ denotes the additive noise signal. When considering clean binaural signals $h_{m}\left(n\right)$ denotes a RIR between the speech source and the M= two eardrums of the listener. In this case $v_{m}\left(n\right)=0\;\forall \;n$ . When considering noisy reverberant signals to be processed by hearing aids, M=6 and $h_{m}\left(n\right)$ denotes a RIR between the speech source and one of the front, middle or back microphone of either left or right hearing aid. In this case $v_{m}\left(n\right)$ is generated from recordings of daily noises, e.g., washing machine, scaled to obtain various signal to noise ratios (SNRs) ranging from −6 to 6 dB. In all cases the reverberant signal $x_{m}\left(n\right)$ is generated using geometric models of rooms with various characteristics using the method described in (Schröder and Vorländer, 2011) and binaural RIRs from (Denk et al., 2018).

### 2.2 Hearing aid processing

Modern hearing aids are typically equipped with multiple microphones whose input is processed to obtain the signal to be played in each ear of the listener. When considering the noisy reverberant signals from the CPC, the M=6 channel signal $y_{m}\left(n\right)$ (3 microphones per hearing aid) was reduced to two channels to be played to the left and right ear. All 20 algorithms considered in this paper were submitted to the clarity enhancement challenge (CEC) (Graetzer et al., 2021), 10 during its first edition (CEC1) and 10 during its second edition (CEC2). This selection of speech enhancement algorithms covers a wide range of approaches, including single-channel source separation, multichannel beamforming and various deep-learning based methods. All algorithms aimed at improving the speech intelligibility of the signals and their performance was evaluated using listening tests. They all aimed at realistic hearing aid applications and used causal signal processing with an algorithm latency of maximum 5 m. Most of these algorithms used the audiogram (see Subsection 2.5) to tailor the processing to each hearing impaired listener in the considered corpus. The same audiograms were used in this paper to simulate the effect of hearing loss.

### 2.3 Hearing loss simulator

The hearing loss simulator aims at simulating the detrimental effect of the hearing loss of each particular listener to the processed signal $z_{m}\left(n\right)$ . The simulator used in this paper relies on the implementation provided as part of the CPC that is based on the well-recognised Cambridge MSBG hearing loss model, named after the authors of the various papers describing it (Moore and Glassber, 1994; Baer and Moore, 1993; 1994; Nejime and Moore, 1997; 1998). This simulator can be briefly described as follows. First, a filter is applied to simulate the acoustic effect of sound propagating to the eardrum before applying spectral smearing to mimic the reduced frequency selectivity of hearing impaired listeners. Then, loudness recruitment simulates the reduced response in the speech frequency range, typical of hearing impairment. A gammatone filterbank is used to extract envelopes at different frequency bands and each envelope is compressed according to the audiogram of the target listener. These compressed envelopes are finally used as gain to adjust the amplitudes of the input signal before resynthetizing the time-domain signal ${\hat{s}}_{m}\left(n\right)$ .

### 2.4 Automatic speech recognition

This paper focuses on the application of ASR in an ARP. Consequently, the chosen ASR system is designed with the limitations typically present in such systems. First, speech is often recorded using a single microphone. Consequently, for each recording, both channels ${\hat{s}}_{1}\left(n\right)$ and ${\hat{s}}_{2}\left(n\right)$ are input separately to the ASR system, as depicted in Figure 2. Additionally, ASR in a ARP often has to rely on models that can be used offline, potentially using hardware of limited capabilities. For this purpose, the Vosk toolkit (Shmyrev, 2023) is chosen in this paper due to its capabilities and its ubiquitousness in robotic applications. The Vosk toolkit provides numerous models for 20 different languages. Four of the available English language models are used in this paper. They are referred to as A, B, C, and D in the remainder of this paper and their full names, sizes and performance using the clean test data from the LibriSpeech (Panayotov et al., 2015) corpus are summarized in Table 1. Larger models are typically expected to yield better performance. It should as well be noted that in order to conform to the requirements of the Vosk toolkit, all signals were downsampled to a sampling frequency of 16 kHz prior to the ASR stage.

**TABLE 1:** Full names and labels of the Vosk models used throughout the paper. Larger models are typically expected to yield better performance, as shown here with their respective size and WER using the LibriSpeech corpus.

	Model	Size	WER [%]
A	small-en-us-0.15	40 M	9.85
B	en-us-0.22-lgraph	128 M	7.82
C	en-us-0.22	1.8 G	5.69
D	en-us-0.42-gigaspeech	2.3 G	5.64

### 2.5 Evaluation

The performance of the ASR system using the four considered models was assessed in terms of (WER) and (WIP) defined in Equations 2, 3, respectively.

$$\text{WER}=100\cdot \frac{S+D+I}{N},$$ (2)

where S , D , I , and N denote the number of substitutions, deletions, insertions and number of words to be recognized, respectively, and the WIP is defined as

$$\text{WIP}=100\cdot \left(\frac{C}{N}+\frac{C}{P}\right),$$ (3)

where C and P , denote the number of correctly recognized words and the number of words in the predicted utterance, respectively. The design of ASR systems aims at a lower WER but a higher WIP. In case of many insertions, WER can be higher than 100%. Both WER and WIP are computed using output from the whole dataset.

**FIGURE 3:**
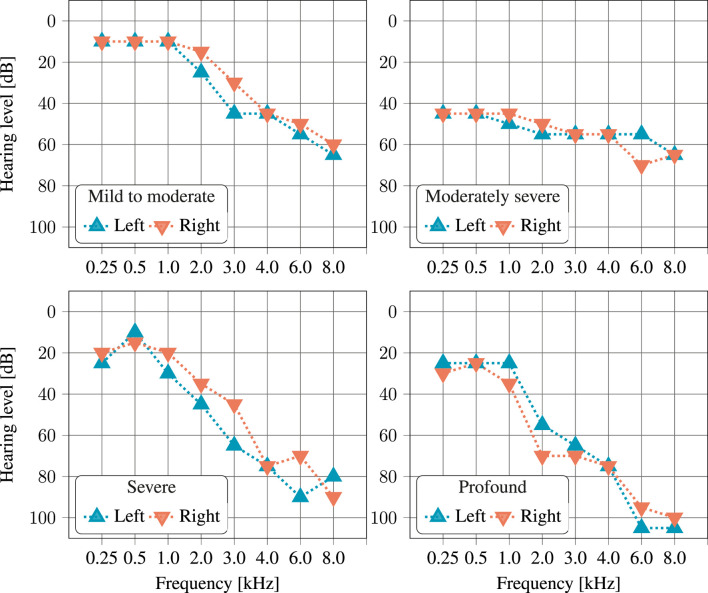
Example of audiograms, left and right ear, for one listener of each of the four considered hearing loss categories. Each of these four examples was randomly selected from the audiograms available in the dataset. The individuality of hearing loss can clearly be seen, though the fact that hearing loss is typically larger at higher frequencies is apparent in all examples.

When reporting WER and WIP for a particular category of hearing loss, it entails applying the previously described methods to the subset of listeners whose audiogram can be fit into this category. In this paper, this was done by averaging the loss over both ears and all frequencies present in the audiogram. The resulting average loss was then categorized according to the scale proposed in (Clark, 1981). Normal to moderate degree of hearing loss (−10–55 dB) were grouped into a single category. Three other categories were considered, namely,: moderately severe (56–70 dB), severe (71–90 dB) and profound ( ≥ 91 dB) hearing loss. The number of listeners per category of hearing loss is depicted in Table 2 and audiogram examples, for each hearing loss category, are depicted in Figure 3. Correlations were reported using the Pearson coefficient ρ and the adequacy of linear fittings were assessed using the coefficient of determination ${\text{R}}^{2}$ .

**TABLE 2:** Number of listeners per category of hearing loss.

Category	Number
Normal to moderate	8
Moderately severe	17
Severe	22
Profound	2
Total	50

## 3 Results

This section presents the performance of the ASR system mentioned above using the four considered models.

First, we observed the performance using the clean binaural signals. Next, we examined the performance using the processed, noisy, reverberant signals. Finally, we studied the relation between the WER from the ASR system and the WER calculated from the responses of human listeners.

### 3.1 Clean binaural signals

**FIGURE 4:**
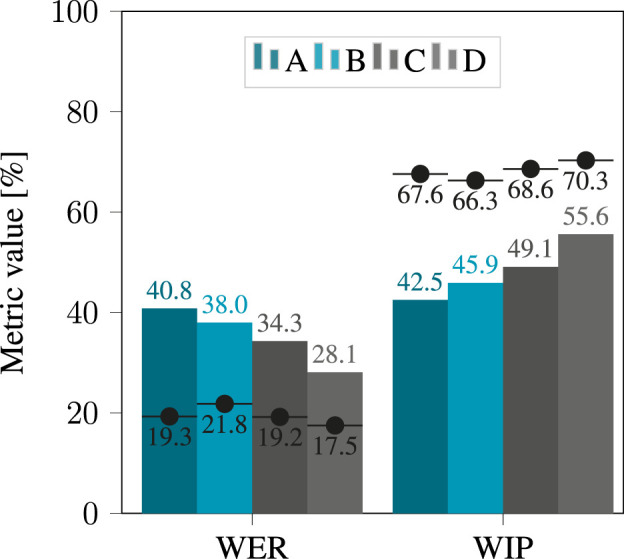
WER and WIP obtained after applying hearing loss simulation to the clean binaural signals, using the four considered models. The black dots denote the score obtained when applying no simulator, i.e., without hearing loss.

The WER for four models using clean binaural signals is shown in Figure 4. The largest model D consistently performs best, whether hearing loss simulation is applied (with a WER of 28.1%) or not (with a WER of 17.5%). The performance of all four models degrades when hearing loss simulation is applied, with the largest difference observed for A, the smallest model, for which the WER degrades from 19.3% to 40.8% when hearing loss simulation is applied. This confirms that the effect of hearing loss simulation, even on clean binaural signals, is detrimental to the performance of ASR systems.

**FIGURE 5:**
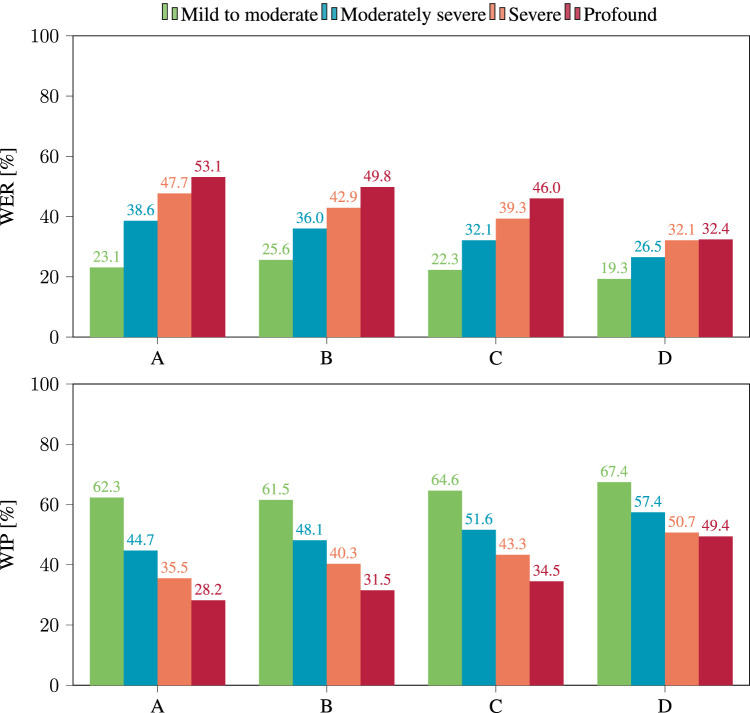
WER (top) and WIP (bottom) per hearing loss category obtained after applying hearing loss simulation to the clean binaural signals, using the four considered models.

The effect of the degree of hearing loss on performance is shown in Figure 5. For all four models, performance declined with increasing severity of hearing loss. Again, the largest discrepancy is found for model A, with a WER of 23.1% for “mild to moderate” hearing loss, increasing to 53.1% for “profound” hearing loss.

For all considered categories of hearing loss, the WER decreases as ASR models get larger, with the minor exception of model A and B in presence of “mild to moderate” hearing loss. In this case, the WER was measured at 23.1% for A and at 25.6% for B. This suggests that model size alone is not always enough to predict the performance of ASR models. It should be noted that overall performance was poor, suggesting that the evaluated corpus could pose a challenge for ASR. Even the best performing model, D, only achieved a WER of 19.3% for moderate hearing loss.

The same trends are seen when analyzing the WIP. Based on the WIP shown in Figure 4, model D performed best with a WIP of 70.3% on unprocessed binaural signals and 55.6% when the hearing loss simulation was applied. For all considered categories of hearing loss, the WIP increased with the size of the ASR model. Looking at the analysis of the effect of hearing loss severity on WIP in Figure 5, it is clear that performance declined with increasing severity for all four models. Again, the most significant contrast was exhibited by model A, which displayed a WIP of 62.3% for a hearing loss categorized as “mild to moderate”, and reduced to 28.2% for a hearing loss categorized as “profound”.

### 3.2 Processed noisy and reverberant signals

**FIGURE 6:**
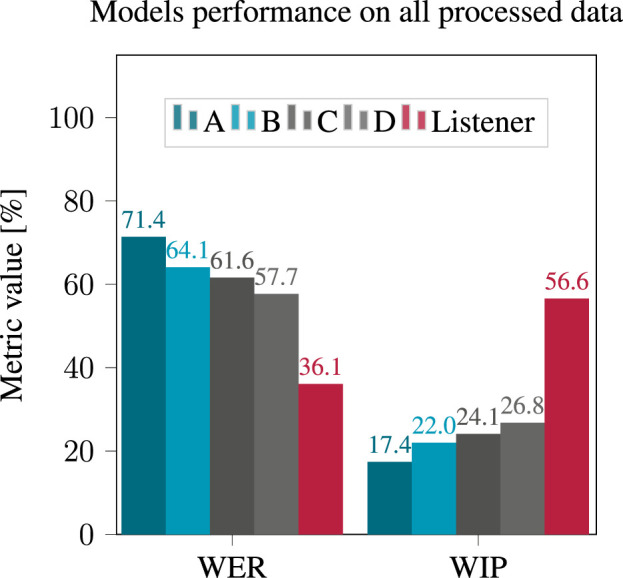
WER and WIP obtained after applying hearing loss simulation to the processed reverberant and noisy signals, using the four considered models.

The WER achieved by the four models under consideration while using processed noisy and reverberant signals is illustrated in Figure 6.

The performance of all models significantly decreased compared to the clean binaural case. Model D (the largest) yielded the best performance with a WER of 57.7% while model A yielded a WER of 71.4%. Due to the high WER, this was interpreted as an unsatisfactory performance of all models rather than a true superiority of model D. The human listeners were able to recognize words much more clearly than any of the models, with a WER of 36.1%.

**FIGURE 7:**
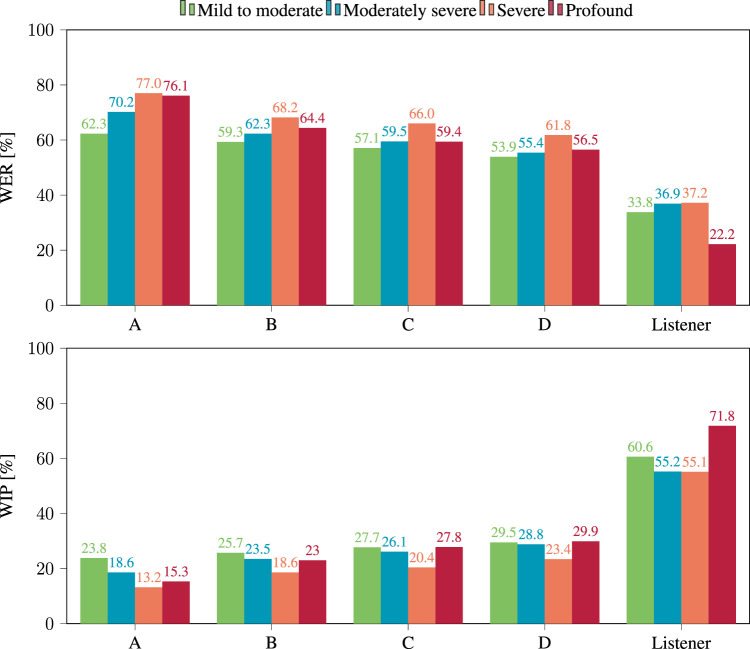
WER (top) and WIP (bottom) per hearing loss category obtained after applying hearing loss simulation to the processed reverberant and noisy signals, using the four considered models.

The effect of hearing loss severity is evident in Figure 7, which demonstrates the degradation of performance across all four models as hearing loss severity increases. When considering the effect on the intelligibility of the listeners, it is noteworthy that the highest WER does not always occur in cases of profound hearing loss, which was unexpected. However, this is most likely an anomaly due to the fact that only two listeners with profound hearing loss are present in the considered dataset (see Table 2).The same trends appeared when considering the WIP. The results achieved by the four examined models using processed noisy and reverberant signals are shown in Figure 6.

In this case, model D yielded the best performance with a WIP of 26.8%, while model A had the lowest WIP at 17.4%. The effect of the severity of the hearing loss on the WIP, as shown in Figure 7, indicated that the WIP decreased as the severity of the hearing loss increased, except in the case of “profound” hearing loss, for which ASR performance appears better then for “moderate” hearing loss.

Examining the recognition performance of human listeners in terms of both WER and WIP as depicted in and Figures 6, 7, similar trends appear but with large difference in absolute value with the performance of ASR models. These findings imply that ASR system performance may not accurately replicate a patient’s performance in the studied situations regarding absolute values of WER or WIP. Even so, it is worth considering the correlation between the performance of ASR systems and WER calculated from the responses of human listeners.

### 3.3 Relation between ASR performance and intelligibility

**FIGURE 8:**
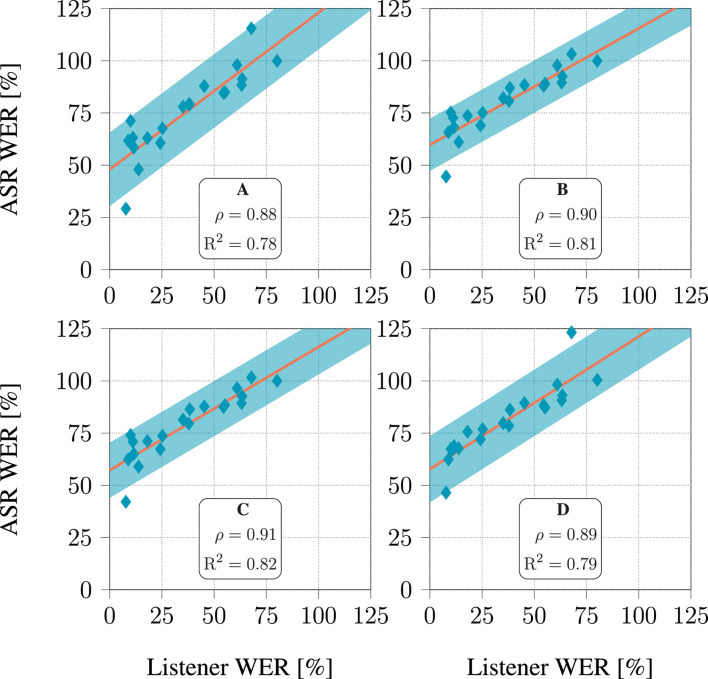
Relationship between WER predicted by ASR models and listener response for each of the 20 hearing aid algorithms in the dataset. For each algorithm, all audiograms available in the dataset were used. The line depicts a linear fit, and the shaded area covers 3 standard deviations above and below this line.

**FIGURE 9:**
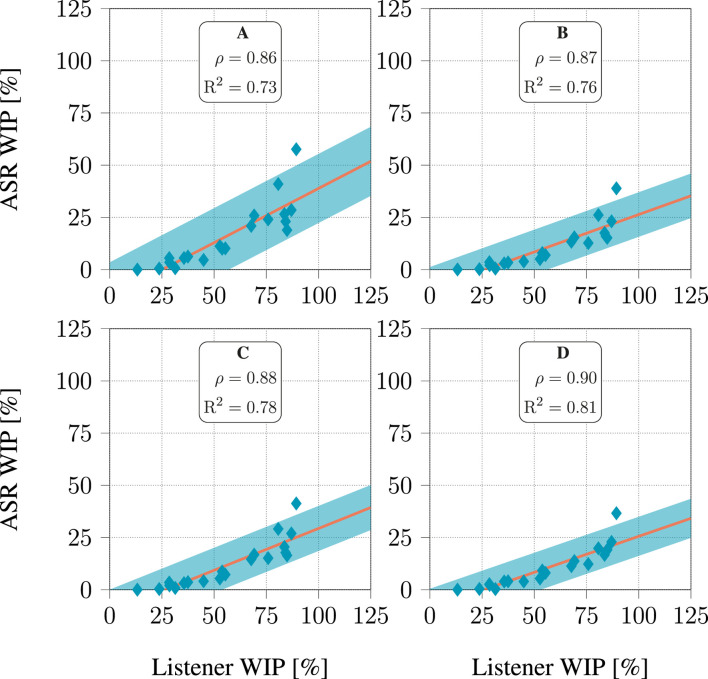
Relationship between WIP predicted by ASR models and listener response for each of the 20 hearing aids algorithms in the dataset. For each algorithm, all audiograms available in the dataset were used. The line represents a linear fit, and the shaded area covers 3 standard deviations above and below this line.

The relationship between the WER and WIP of the ASR system and those derived from human listeners’ responses are presented in Figures 8, 9, respectively. The human listeners (see Table 2) had to recognize the speech from the signals processed with hearing aid algorithms as part of the challenge evaluation (Barker et al., 2022; Graetzer et al., 2022). Figures 8, 9 depict, the value of these metrics obtained when considering the signals processed with each of the 20 hearing aid processing algorithms included in the dataset. Each listener had to listen to a few hours of processed speech. It seems that both WER and WIP for all four models displayed a high correlation with those computed from the listeners’ responses, with ρ ranging from 0.88 to 0.96 when considering WER, and from 0.85 to 0.94 when considering WIP. Furthermore, it was evident that the correlation could be precisely depicted through linear regression, as indicated by the high ${\text{R}}^{2}$ coefficient values ranging from 0.78 to 0.91 when considering WER, and from 0.73 to 0.88 when considering WIP. A hearing aid processing algorithm consistently produced results that did not match the linear relationship. This is the algorithm described in (Cornell et al., 2023), which was the most successful algorithm during the CEC2.

## 4 Conclusion

The simulation of disease-specific patient behavior by ARP will become increasingly important in the following years. Details such as the patient’s hearing loss are critical to achieving the correct ARP behavior for realistic training and education of medical staff. Therefore, the effects of hearing loss and hearing enhancement algorithms on ASR systems were evaluated in this paper.

Experiments were conducted using both clean binaural signals and noisy reverberant signals processed using hearing aids speech enhancement algorithms. The impact of hearing loss was simulated using audiograms measured on real human listeners. All data is available as part of the CPC and the ASR transcription compared publicly available models to be used with the Vosk toolkit. The performance of these different Vosk models was evaluated using WER and WIP.

In the initial experiment, using binaural signal with and without applying hearing loss simulation, the largest considered model outperformed all other models, with the smallest model coming in second place. Notably, all models yielded lower performance in presence of hearing loss simulation. When the hearing loss simulation was applied to processed, reverberant, and noisy signals, all four models performed worse than human listeners. The biggest model performed best. Furthermore, a strong correlation was observed between the WER and WIP of all four models and the responses of the listeners. Therefore, it can be concluded that the hearing loss simulation significantly impacts ASR. Moreover, it appears that the size of the models did not play a significant role in this experiment, as with increasing model size the performance did not increase accordingly. Nevertheless, the biggest model outperformed the smaller models.

Aiming to use data that was both realistic and publicly available, all results were obtained using the data from the Clarity Challenge dataset. However, this choice does come with some limitations. This dataset does not include reverberant conditions without the use of hearing aid algorithms or the recognition scores of the listeners to the clean data, which would be beneficial for future experiments. Additionally, though realistic, the text content of this dataset was not designed specifically for patient simulation, i.e., the text content has no relation to the patient simulation that motivates this paper, which could be a future target. Furthermore, a dataset of speech utterances would allow future work to use clean unprocessed speech that could as well be used to generate speech under various acoustic conditions. Of course, this will as well allow us to extend the evaluation considering speech better matching the target use case of the ARP.

Focusing on the future use of ARPs for medical education and verbal medical assessments, clinical background noise, weak voices, and the choice of words used in the assessment should be considered in following work.

## Data Availability

Publicly available datasets were analyzed in this study. This data can be found here: https://claritychallenge.org/docs/cpc2/cpc2_intro.

## References

[B1] Abe S. Noguchi N. Matsuka Y. Shinohara C. Kimura T. Oka K. (2018). Educational effects using a robot patient simulation system for development of clinical attitude. Eur. J. Dent. Educ. 22, e327–e336. 10.1111/eje.12298 29091328

[B2] Association A. P. Association A. P. (2013). Diagnostic and statistical manual of mental disorders: dsm-5. 5th ed. edn. Arlington, VA: American Psychiatric Association.

[B3] Austin Z. Gregory P. Tabak D. (2006). Simulated patients vs. standardized patients in objective structured clinical examinations. Am. J. Pharm. Educ. 70, 119. 10.1016/s0002-9459(24)07776-3 17149448 PMC1636998

[B4] Baer T. Moore B. C. (1993). Effects of spectral smearing on the intelligibility of sentences in noise. J. Acoust. Soc. Am. 94, 1229–1241. 10.1121/1.408176 8201124

[B5] Baer T. Moore B. C. (1994). Effects of spectral smearing on the intelligibility of sentences in the presence of interfering speech. J. Acoust. Soc. Am. 95, 2277–2280. 10.1121/1.408640 8201124

[B6] Baiduc R. R. Sun J. W. Berry C. M. Anderson M. Vance E. A. (2023). Relationship of cardiovascular disease risk and hearing loss in a clinical population. Sci. Rep. 13, 1642. 10.1038/s41598-023-28599-9 36717643 PMC9886989

[B7] Barker J. Akeroyd M. Cox T. J. Culling J. F. Firth J. Graetzer S. (2022). The 1st Clarity Prediction Challenge: a machine learning challenge for hearing aid intelligibility prediction. Proc. Interspeech, 3508–3512. 10.21437/Interspeech.2022-10821

[B8] Barrows H. S. (1968). Simulated patients in medical teaching. Can. Med. Assoc. J. 98, 674–676. 5646104 PMC1924019

[B9] Buchanan J. A. (2001). Use of simulation technology in dental education. J. Dent. Educ. 65, 1225–1231. 10.1002/j.0022-0337.2001.65.11.tb03481.x 11765868

[B10] Buskens E. Vogt T. C. Liefbroer A. C. Reijneveld M. S. Bultmann U. Henkens K. C. (2019). Healthy ageing: challenges and opportunities of demographic and societal transitions. Older People Improv. Health Soc. Care Focus Eur. Core Competences Framew., 9–31. 10.1007/978-3-319-97610-5_2

[B11] Cae I. (2022). Cae apollo. Date last (Accessed September 20, 2022).

[B12] Clark J. G. (1981). Uses and abuses of hearing loss classification. Asha 23, 493–500. 7052898

[B13] Cleland J. A. Abe K. Rethans J.-J. (2009). The use of simulated patients in medical education: amee guide no 42. Med. Teach. 31, 477–486. 10.1080/01421590903002821 19811162

[B14] Cornell S. Wang Z.-Q. Masuyama Y. Watanabe S. Pariente M. Ono N. (2023). Multi-channel target speaker extraction with refinement: the wavlab submission to the second clarity enhancement challenge

[B15] Dalton D. S. Cruickshanks K. J. Klein B. E. Klein R. Wiley T. L. Nondahl D. M. (2003). The impact of hearing loss on quality of life in older adults. gerontologist 43, 661–668. 10.1093/geront/43.5.661 14570962

[B16] [Dataset] Gaumard Scientific Company, I (2022a). Hal tetherless simulators (Accessed September 20, 2022).

[B17] [Dataset] Gaumard Scientific Company, I. (2022b). Susie tetherless simulators . (Last accessed September/20/2022)

[B18] Denk F. Ernst S. M. Ewert S. D. Kollmeier B. (2018). Adapting hearing devices to the individual ear acoustics: database and target response correction functions for various device styles. Trends Hear. 22, 233121651877931. 10.1177/2331216518779313 PMC5992802 29877161

[B19] Devlin J. W. Skrobik Y. Gélinas C. Needham D. M. Slooter A. J. Pandharipande P. P. (2018). Clinical practice guidelines for the prevention and management of pain, agitation/sedation, delirium, immobility, and sleep disruption in adult patients in the icu. Crit. care Med. 46, e825–e873. 10.1097/ccm.0000000000003299 30113379

[B20] Ely E. Gautam S. Margolin R. Francis J. May L. Speroff T. (2001a). The impact of delirium in the intensive care unit on hospital length of stay. Intensive care Med. 27, 1892–1900. 10.1007/s00134-001-1132-2 11797025 PMC7095464

[B21] Ely E. W. Margolin R. Francis J. May L. Truman B. Dittus R. (2001b). Evaluation of delirium in critically ill patients: validation of the confusion assessment method for the intensive care unit (cam-icu). Crit. care Med. 29, 1370–1379. 10.1097/00003246-200107000-00012 11445689

[B22] Ely E. W. Shintani A. Truman B. Speroff T. Gordon S. M. Harrell Jr F. E. (2004). Delirium as a predictor of mortality in mechanically ventilated patients in the intensive care unit. Jama 291, 1753–1762. 10.1001/jama.291.14.1753 15082703

[B23] Fadel W. Araf I. Bouchentouf T. Buvet P.-A. Bourzeix F. Bourja O. (2022). “Which French speech recognition system for assistant robots?,” in 2022 2nd international conference on innovative research in applied science, engineering and Technology (IRASET) (IEEE), 1–5.

[B24] Gaba D. M. (2004). The future vision of simulation in health care. BMJ Qual. and Saf. 13, i2–i10. 10.1136/qhc.13.suppl_1.i2 PMC1765792 15465951

[B25] Glauser R. Holm J. Bender M. Bürkle T. (2023). How can social robot use cases in healthcare be pushed-with an interoperable programming interface. BMC Med. Inf. Decis. Mak. 23, 1–11. 10.1186/s12911-023-02210-7 PMC10337241 37434236

[B26] Graetzer S. Akeroyd M. A. Barker J. Cox T. J. Culling J. F. Naylor G. (2022). Dataset of british English speech recordings for psychoacoustics and speech processing research: the clarity speech corpus. Data Brief 41, 107951. 10.1016/j.dib.2022.107951 35242933 PMC8881678

[B27] Graetzer S. Barker J. Cox T. J. Akeroyd M. Culling J. F. Naylor G. (2021). Clarity-2021 challenges: machine learning challenges for advancing hearing aid processing. Proc. Interspeech 2, 686–690. 10.21437/Interspeech.2021-1574

[B28] Gu D. Andreev K. Dupre M. E. (2021). Major trends in population growth around the world. China CDC Wkly. 3, 604–613. 10.46234/ccdcw2021.160 34594946 PMC8393076

[B29] Guenther U. Popp J. Koecher L. Muders T. Wrigge H. Ely E. W. (2010). Validity and reliability of the cam-icu flowsheet to diagnose delirium in surgical icu patients. J. Crit. care 25, 144–151. 10.1016/j.jcrc.2009.08.005 19828283

[B30] Haley B. Heo S. Wright P. Barone C. Rettigantid M. R. Anders M. (2017). Effects of using an advancing care excellence for seniors simulation scenario on nursing student empathy: a randomized controlled trial. Clin. Simul. Nurs. 13, 511–519. 10.1016/j.ecns.2017.06.003

[B31] Hashimoto T. Nakane H. Kobayashi H. (2013). “Android patient robot simulating depressed patients for diagnosis training of psychiatric trainees,” in 2013 second international Conference on robot, Vision and signal processing (IEEE), 247–252.

[B32] Moore B. C. Glassber B. R. (1994). Simulation of the effects of loudness recruitment and threshold elevation on the intelligibility of speech in quiet and in a background of speech. J. Acoust. Soc. Am. 94, 2050–2062. 10.1121/1.407478 8227747

[B33] Morandi A. Inzitari M. Udina C. Gual N. Mota M. Tassistro E. (2021). Visual and hearing impairment are associated with delirium in hospitalized patients: results of a multisite prevalence study. J. Am. Med. Dir. Assoc. 22, 1162–1167.e3. 10.1016/j.jamda.2020.09.032 33160873

[B34] Nejime Y. Moore B. C. (1997). Simulation of the effect of threshold elevation and loudness recruitment combined with reduced frequency selectivity on the intelligibility of speech in noise. J. Acoust. Soc. Am. 102, 603–615. 10.1121/1.419733 9228821

[B35] Nejime Y. Moore B. C. (1998). Evaluation of the effect of speech-rate slowing on speech intelligibility in noise using a simulation of cochlear hearing loss. J. Acoust. Soc. Am. 103, 572–576. 10.1121/1.421123 9440342

[B36] Panayotov V. Chen G. Povey D. Khudanpur S. (2015). “LibriSpeech: an ASR corpus based on public domain audio books,” in Proc. IEEE intl. Conf. On acoustics, Speech and signal processing (ICASSP) (South brisbane, QLD, Australia), 5206–5210.

[B37] Paul S. Sintek M. Silaghi M. Këpuska V. Robertson L. (2022). “A novel multimodal situated spoken dialog system for human robot communication in emergency evacuation,” in 2022 21st IEEE international conference on machine learning and applications (ICMLA) (IEEE), 1660–1665.

[B38] Pourebadi M. Riek L. D. (2022). Facial expression modeling and synthesis for patient simulator systems: past, present, and future. ACM Trans. Comput. Healthc. 3, 1–32. 10.1145/3483598

[B39] Röhl J. H. Hellmers S. Diekmann R. Hein A. (2022). “Concept of an observation-driven android robot-patient with individualized communication skills,” in 2022 9th IEEE RAS/EMBS international conference for biomedical robotics and biomechatronics (BioRob) (IEEE), 1–7.

[B40] Röhl J. H. Klausen A. D. Feldmann N. Diekmann R. Hellmers S. Günther U. (2023). “Android robot-patient for teaching and training of delirium assessment instruments: a pilot study,” in 2023 IEEE international conference on advanced robotics and its social impacts (ARSO) (IEEE), 78–83.

[B41] Rudolph J. L. Inouye S. K. Jones R. N. Yang F. M. Fong T. G. Levkoff S. E. (2010). Delirium: an independent predictor of functional decline after cardiac surgery. J. Am. Geriatrics Soc. 58, 643–649. 10.1111/j.1532-5415.2010.02762.x PMC2856754 20345866

[B42] Rudolph J. L. Jones R. N. Levkoff S. E. Rockett C. Inouye S. K. Sellke F. W. (2009). Derivation and validation of a preoperative prediction rule for delirium after cardiac surgery. Circulation 119, 229–236. 10.1161/circulationaha.108.795260 19118253 PMC2735244

[B43] Schröder D. Vorländer M. (2011). “RAVEN: a real-time framework for the auralization of interactive virtual environments,” in *Forum acusticum* (aalborg, Denmark), 1541–1546.

[B44] Schwarz P. Hein A. (2023). “Conception of a humanoid-robot-patient in education to train and practice,” in 2023 IEEE 2nd German education conference (GECon) (IEEE), 1–5.

[B45] Shmyrev N. V. (2023). Vosk speech recognition toolkit: offline speech recognition API for android, iOS, Raspberry Pi and servers with Python, Java, C# and Node. Available at: https://github.com/alphacep/vosk-api.

[B46] Tanzawa T. Futaki K. Kurabayashi H. Goto K. Yoshihama Y. Hasegawa T. (2013). Medical emergency education using a robot patient in a dental setting. Eur. J. Dent. Educ. 17, e114–e119. 10.1111/j.1600-0579.2012.00770.x 23279398

[B47] Tanzawa T. Futaki K. Tani C. Hasegawa T. Yamamoto M. Miyazaki T. (2012). Introduction of a robot patient into dental education. Eur. J. Dent. Educ. 16, e195–e199. 10.1111/j.1600-0579.2011.00697.x 22251346

[B48] Tengiz F. İ. Sezer H. Başer A. Şahin H. (2022). Can patient-physician interview skills be implemented with peer simulated patients? Med. Educ. Online 27, 2045670. 10.1080/10872981.2022.2045670 35232322 PMC8896181

